# Evaluation of the expression of integrins and cell adhesion molecules through tissue microarray in lymph node metastases of prostate cancer

**DOI:** 10.4103/1477-3163.48453

**Published:** 2009-02-25

**Authors:** José Pontes-Júnior, Sabrina Thalita Reis, Marcos Dall'Oglio, Luis Carlos Neves de Oliveira, Jose Cury, Paulo Afonso Carvalho, Leopoldo Alves Ribeiro-Filho, Kátia Ramos Moreira Leite, Miguel Srougi

**Affiliations:** Laboratory of Medical Investigation – LIM 55, Urology Department, Medical School University of São Paulo, São Paulo, Brazil

**Keywords:** Cell adhesion molecules, integrins, lymph nodes, metastases, prostate cancer

## Abstract

**Background:**

Integrins and adhesion molecules are responsible for the maintenance of the epithelial phenotype. Cell culture studies have reported the correlation between adhesion molecule expression and prostate carcinoma, but their role in the metastatic process is not yet known. Our aim is to study the expression profiles of these molecules and evaluate their association with the metastatic behavior of prostate adenocarcinoma.

**Materials and Methods:**

A Tissue Microarray containing two samples from 19 primary tumors and one from their corresponding lymph node metastases was constructed and subjected to immunohistochemical analysis of the expression of integrins, E-cadherin and β and γ-catenins. Within each case, paired analyses were also performed to evaluate gains or losses in metastasis compared to its primary tumor.

**Results:**

The expression of αv, αvβ3, α2β1 and γ-catenin were abnormal in almost every case. Marked loss of E-cadherin and β4 integrin was found in primary and metastatic lesions. β-catenin was normal in all primary cases and in 94% of metastases. α6 was normal in all primary tumors and metastases. α3 and α3β1 were normal in 32% of primary cases and in 53% and 6% of metastases, respectively. In paired analyses, loss of E-cadherin, β4, αv, α3β1 and αvβ3 was found in 65%, 71%, 59%, 53% and 47% of patients, respectively. Catenins and α2β1 showed maintenance of expression in most of the cases.

**Conclusions:**

In this preliminary study we have shown that the loss of cell adhesion molecules can be considered a characteristic of the metastatic phenotype in prostate cancer. Larger series should be evaluated in order to confirm our findings.

## Introduction

In normal prostate development, the interaction of glandular epithelial cells with the extracellular matrix is essential; this interaction influences these cells' growth, survival and differentiation. The cell adhesion molecules (CAM) collaborate in this interaction and are responsible for the maintenance of a normal tissue phenotype.

This interaction is also believed to play an important role in oncogenesis, where tumor invasion and progression to metastasis are the signatures of malignancy. Many aspects of oncogenesis involve changes in the adhesion of cells to adjacent cells and to the extracellular matrix. Essentially, cells that adhere well do not metastasize, while less adhesive cells are more likely to do so.

Before dissemination and colonization at a metastatic site, prostate cancer cells must become motile and detach from the primary tumor and overcome the extracellular matrix. This increase in cell motility is accompanied by changes in the expression of adhesion receptors, especially those of the integrin and cadherin families.[1] Cell motility is the result of a coordinated balance between the adhesion and detachment of cells through CAMs, and this property changes simultaneously with tumor cell-induced remodeling of a cell's extracellular matrix.[2] CAMs are divided into four groups: integrins, cadherins, immunoglobulins and selectins.

The cell surface receptor integrins have been implicated as the main participants in these events, since they mediate homotypic (cell to cell) and heterotypic (cell to extracellular matrix) interactions of prostate cancer cells within their microenvironment. Cadherins and catenins, on the other hand, mediate only homotypic interactions.

Integrins are members of a family of transmembrane glycoprotein receptors that regulate cell-matrix and cell-cell interactions. They form heterodimers composed of α and β subunits, with each combination having its own binding specificity and signaling properties.

Currently, 18 α and 8 β subunits have been identified, and different combinations of α and β subunits dictate their specificity for extracellular ligands.[3] The mechanism involved in coordinating the heterodimer usage by cells has not been determined. Each integrin subunit has a large extracellular domain and a transmembrane stretch, thus, integrins represent the connection between the extracellular environment and the intracellular compartment. As receptors, integrins mediate the anchoring and migration of cells via recognition of variable extracellular matrix molecules.[2] Moreover, intracellular signals generated by integrins influence gene expression and affect the regulation of cell survival, differentiation and proliferation.[4] In normal prostate basal cells, E-cadherin, catenins and several integrins are normally expressed, including α2 to 6, αv, β1 and β4.

It is believed that the changes in integrin expression that occur during malignant transformation are highly dependent on the type of the neoplasm and that these changes allow the cancer cells to recognize variable matrices and influence adhesion, migration, signaling and gene expression.[5,6]

In prostate cancer, only limited observations related to integrin expression in prostate cancer progression have been made due to a paucity of *in vivo* immunohistochemical studies in metastatic disease. Most of our current knowledge in the metastatic setting is derived from cell lines' studies.[7,8] *In vitro* studies of prostate cancer progression have revealed changes in integrin expression, but it remains unknown whether these observations can be translated to *in vivo* processes.

E-cadherin is a calcium-dependent transmembrane glycoprotein that plays a role in cellular adhesion. Its intracellular domain connects to catenins (α, β and γ) in the cell cytoplasm, which, in turn, are physically associated with the actin filaments of the cytoskeleton. This extracellular domain accounts for the role of E-cadherins in homotypic intercellular adhesion. The proper interaction between E-cadherins and catenins is essential to the maintenance of epithelial cell integrity and a benign epithelial phenotype.[9,10]

The aim of our study is to assess the expression of integrins and other CAMs in primary and lymph node metastasis of prostate cancer by immunohistochemistry using a Tissue Microarray (TMA) technique.

## Materials snd Methods

### Case selection

Between March 1997 and July 2006, 1619 patients underwent radical prostatectomy and iliac lymphadenectomy by the same surgeon (MS). In 19 patients the lymph nodes were positive for metastatic cancer and all these cases were selected for this study; however, in two cases the tumor was scarce and insufficient for immunohistochemical analysis. The slides containing the metastasis and the primary tumor for each patient were selected by considering the area that best represented the whole tumor. One area from the metastasis and two areas from the primary tumor were selected and marked with permanent ink; these areas correspond to those included in the TMA. This study conforms to the provisions of the Declaration of Helsinki and was submitted to and approved by the Ethical Board of the HCFMUSP under the protocol 1074/04.

### Immunohistochemistry

**Figure 1:**
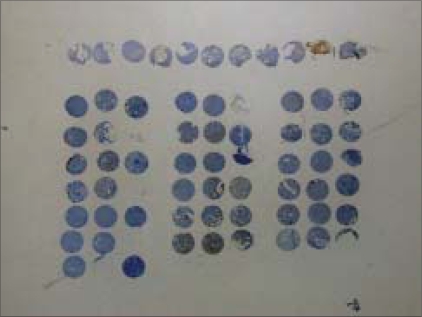
Tissue Microarray containing, for each case, two samples from the primary tumor and one from the lymph node

The TMA was constructed on one superfrost slide containing two samples from the primary tumor and one from lymph node metastasis [Figure 1].

In a heat antigen retrieval process, the slides were placed in a citrate buffer (1 mM, pH 6.0) and heated for 30 min in the steamer. The slides were incubated overnight at 4 °C with the monoclonal antibodies specified in Table 1. The LSAB system was used for immunostaining (LSAB; Dako Cytomation, CA, USA). Color was developed by reaction with a 3,3'diaminobenzidine substrate-chromogen solution followed by counterstaining with Harris hematoxylin. Slides were dehydrated, coverslipped and observed under the light microscope. Semiquantitative analysis was performed for all antibodies, and the expression intensity was divided into three groups: weak: +, moderate: ++ and strong: +++. Distribution of staining was divided into two groups: focal or diffuse. Only strong and diffuse expression (i.e., positive staining +++ in more than 70% of cells), as described by others,[11–15] were considered normal. The expression was classified as abnormal if the intensity of staining was negative, weak or moderate, or if the staining was focal.

**Table 1:** Antibodies and their respective dilutions

Antibody	Manufacturer	Dilution
γ-catenin	Zymed (San Francisco, CA, USA)	1:100
β-catenin	BD (San Jose, CA USA)	1:50
E-cadherin	Dako (Dako Cytomation, CA, USA)	1:50
Av	Calbiochem (San Diego CA, USA)	1:2000
α3β1	Chemicon (Temecula CA, USA)	1:100
α3	Chemicon (Temecula CA, USA)	1:100
β4	Chemicon (Temecula CA, USA)	1:100
α6	Abcam (Cambridge, MA, USA)	1:200
αvβ3	Abcam (Cambridge, MA, USA)	1:50
α2β1	Chemicon (Temecula CA, USA)	1:100

The expression of each marker in the primary tumor and lymph node was evaluated by one pathologist (KRML). For statistical analyses, the differences in expression for both sites were evaluated by the Fisher Exact Test.

The analysis of both primary and metastatic tumor for the same patient was possible in 17 cases; for those we compared the expression for all markers in both specimens intending to characterize and understand the pathways that lead to progression of prostate cancer. We assume that the detection of gain or loss of a marker is important since it may reflect some of the characteristics that may promote tumor progression. For this analysis we recorded any degree of gain or loss of expression and changes in the distribution of staining.

## Results

The mean age of patients was 66 years, and the mean follow-up time was 3.4 years. In six cases, there was no information about previous treatments or follow-ups after surgery. None of the remaining patients with metastatic prostate cancer received radiotherapy or chemotherapy prior to surgery, and only one patient received neoadjuvant androgen deprivation therapy. The pre-surgical PSA was available in all cases; the mean serum PSA value was 11.9 ng/mL, and the median was 8.8 ng/mL (ranging from 4.6 to 44.0 ng/mL). The Gleason score of the surgery specimens was 10 in three cases, 9 in ten cases, 8 in five cases and 7 in one case. Sixteen patients were staged pT3b, two were pT3a and one was pT2c. The mean tumor volume was 45 cm^3^ and the median tumor volume was 36 cm^3^ (ranging from 7 to 100 cm^3^).

After surgery, 12 out of 13 patients initiated adjuvant androgen deprivation therapy because of lymph node metastasis, and only one developed a hormone refractory disease. The patient who did not receive hormone therapy had no sign of biochemical recurrence at a follow-up 3.5 years later.

Immunohistochemical analysis was impossible in two lymph node metastases due to limited tissue available. The results of E-cadherin, catenins and integrins expression in the 19 primary tumors and 17 lymph node metastases are given in Table 2.

**Table 2:** Immunoexpression of E-cadherin, catenins and integrins in 19 primary and 17 lymph node metastases of prostate adenocarcinoma

	Primary tumor Expression (%)		Lymph node Expression (%)		*P* value	
			
	normal	abnormal	normal	abnormal	
γ-catenin	5	95	12	88	0,593
β-catenin	100	0	94	6	0,472
E-cadherin	11	89	29	71	0,219
α v	0	100	0	100	-
α3β1	32	68	6	94	0,092
α3	32	68	53	47	0,311
β4	16	84	6	94	0,605
α6	100	0	100	0	-
αvβ3	0	100	0	100	-
α2β1	0	100	0	100	-

In the primary tumors, αv, αvβ3, α2β1 and γ-catenin showed abnormal expression in almost every case; similar results were found in the metastases for these same markers.

Conversely, β-catenin and α6 showed normal expression in all primary cases and in most of the metastases. α3 and α3β1 expression was normal in 32% of primary cases and in 53% and 6% of metastases, respectively.

E-cadherin and β4 expression was normal in only 11% and 16% of primary tumors and in 29% and 6% of the metastatic sites, respectively.

In the majority of cases, the expression of all antibodies was lower in lymph nodes' metastases than in primary tumors. However, using the Fisher Exact Test, we found no significant difference in the immunoexpression of any of the markers. It was interesting to note that for α3β1, there was a marginally significant difference between the expressions in the primary tumor and those in the metastatic tumor (*P*=0.092).

In the subset of 17 paired cases, we compared the expression for all markers in the primary prostate adenocarcinoma with its respective metastatic tumor. The results are shown in Table 3.

**Table 3:** Gains or losses of immunoexpression of cell adhesion molecules in 17 lymph node metastases compared to their respective primary prostate tumor

	Gain %	Loss %
γ-catenin	18	23
β catenin	0	6
E-cadherin	29	65
αv	6	59
α3β1	12	53
α3	35	18
β4	6	71
α6	0	0
αvβ3	0	47
α2β1	6	29

**Figure 2:**
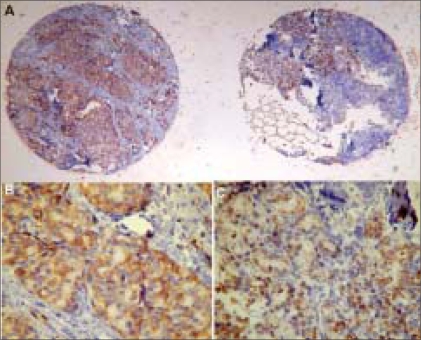
Primary and metastatic lesions of the same patient showing reduction in β4 expression in lymph node metastasis when compared to the primary tuPaired case: primary tumor (left side) and metastasis to lymph node (right side) Primary tumor Lymph node metastasis

**Figure 3:**
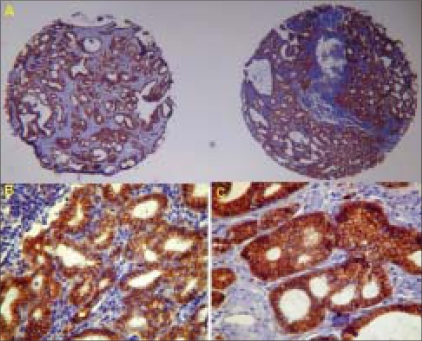
Primary and metastatic lesions of the same patient showing maintenance of β-catenin expression in lymph node metastasis when compared to the primary tumor a) Paired case: primary tumor (left side) and metastasis to lymph node (right side) b) Primary tumor Lymph node metastasis

Comparing metastatic to primary tumors, case by case, we found loss of expression for E-cadherin, β4, αv, αvβ3 and α3β1 in 65%, 71%, 59%, 47% and 53% of patients, respectively. Figure 2 shows a loss of expression of β4 in a paired case. β-catenin and α6 showed maintenance of normal expression in most of the cases. α2β1 and γ-catenin tended to show lower percentages of normal expression in both sites. In Figure 3, we show an example of the maintenance of β-catenin expression in a paired case. Consistent gain of expression was found only for α3 in 35% of cases.

## Discussion

One of the most studied areas in oncology has been the role of CAM and cell-extracellular matrix interactions. It is accepted that CAM plays a crucial role in cell differentiation, growth, survival, proliferation, migration and invasion.[16]

Despite this notion, currently, little is known about the changes of integrin expression in prostate cancer, especially in the metastatic setting. The aim of our study is to describe a profile of CAMs expression in prostate cancer with lymph node metastasis. With the exception of β-catenin and α6, we found a global loss of CAM expression, suggesting that these changes play a role in prostate cancer progression.

In literature, alterations in integrin expression have been documented in primary prostate tumors and prostate cancer cell lines when compared to normal prostate tissue.[17] In primary prostate cancer, the majority of the integrin subunits are already lost, which is consistent with the concept that down-regulation of integrins is related to carcinogenesis in humans.[1,18] In general, the expression of α2, α4, α5, β4 and α6β4 integrins is reduced in prostate cancer, while α3β1 and α6β1 expression is maintained.[18–20]

Nowadays, with the disseminated use of PSA for screening of prostate cancer, few patients submitted to surgery have metastasis to lymph nodes. This fact is reflected in this series with a small number of cases studied.

In our series we observed normal α6 expression in all primary and metastatic tumors and consistently reduced β4 expression in primary and lymph node metastatic tumors. The comparisons between primary and metastatic tumors showed loss of β4 expression in 71% of metastases. It is well known that α6 integrin can pair with either the β1 or β4 subunit and that both will bind to laminin. In normal prostate glands and intraepithelial neoplasia, the β4 subunit is the dominant pairing unit for α6, resulting in the integrin α6β4.[13] α6β4 is an essential integrin for epithelial cell phenotype maintenance, and is necessary for the formation of the hemidesmosome, a structure that acts as a brake to cell migration and proliferation.[21] Loss of the hemidesmosome would result in a less stable interaction of the cell with the extracellular matrix, thus providing an increased potential for tumor cells to invade and metastasize.

It has been hypothesized that during prostate cancer progression the β4 subunit is lost, so the α6 integrin is paired preferentially with the β1 subunit, resulting in α6β1, an integrin that promotes focal contact.[13,18,21] The consequence of this change is exemplified by the shift from a static cell attachment promoted by α6β4 integrin expression in LNCaP cells to a motility state associated with the α6β1 integrin expressed by the highly aggressive C4-2 cell line.[22] In LNCaP cells, α6β4 is important for attachment and restricts cell migration, while α6β1 and α3β1, both of which are involved in the formation of dynamic focal contacts, are important for migration.[23]

This switch from α6β4 to α6β1 was also confirmed by Nagle *et al.*, who used electron microscopy to show the lack of hemidesmosomes during prostate cancer progression.[24] This morphological data was also confirmed by a DNA microarray study that showed loss of expression of β4 in human prostate carcinoma.[25]

Our data support the notion that loss of expression of β4 and maintenance of α6 expression are both related to tumorigenesis and metastatization in prostate cancer, confirming prior findings in primary tumor and cell line studies.[13,22]

There is little information about the role of integrins as prognostic markers in prostate carcinoma. Schmelz *et al.*, studying 135 biopsies containing prostate cancer, found an absence of β4 expression and positive expression for α3 or α6 in 80% of cases. They also reported an inverse correlation between Gleason scores and α3 and α6 expression. In addition, they showed that the clustered pattern of α6 expression was correlated with invasion, indicating that these integrins are possible prognostic markers in prostate cancer.[26]

In some neoplasms, α2β1 expression has been associated with tumor progression and metastasis.[27] Bonkhoff *et al.*, evaluated 33 prostatectomy specimens and 10 metastatic lymph nodes using immunohistochemical.[28] They found preserved or increased α6 expression and down-regulation of α2 expression in low-grade prostate cancer and decreased expression for both in high-grade tumors. Interestingly, increased expression of α6 and α2 was found in the majority of lymph node metastases, leading the authors to conclude that up-regulation of these receptors may contribute to their invasive and metastatic potential in prostate cancer.[28] A study with prostate cancer cell lines also found that α2 expression in the metastatic cell lines was double that in LNCaP cells.[22]

In our study, we showed maintenance of α6 and loss of α2b1 expression in all primary cases, a result that is in accordance with the report of Bonkhoff;[28] but we could not confirm super-expression of α2β1 in the metastatic lesions.

The functional role of α3 is still poorly understood; we observed normal α3 expression in one-third of primary cases and in half of metastases, illustrating that an increase in α3 expression could be related to tumor progression. In paired analyses, α3 was the integrin with the higher gain of expression in lymph nodes.

As previously discussed, α3β1 and α6β1 are both involved in the formation of dynamic focal contact important for cell motion.[23] This is in contrast to α6β4, which is associated with restricted cell migration. This led us to conclude that α3, together with α6, may be important for giving cells motility and allowing prostate cancer dissemination.

αvβ3 is a candidate integrin for assisting in metastasis of prostate cancer to bone.[29,30] Prostate cancer cell adhesion and migration in dominant components of the bone matrix, such as osteopontin and vitronectin, are mediated by αvβ3.[31] Antibodies blocking αvβ3 integrin have been shown to reduce prostate cancer cell adhesion to bone protein extracts by 94%.[31] Edlund *et al.*, showed that LNCaP cells had undetectable levels of αvβ3 but that metastatic-derived cells from LNCaP cells showed consistent expression of this integrin. They also showed that an antibody against αvβ3 inhibited cell migration on vitronectin and osteopontin.[22] However, in our series, αvβ3 and αv were under-expressed in all primary tumors and their lymph node metastasis.

We have shown the loss of E-cadherin expression in 89% of primary and 71% of metastatic tumors. When we compared the primary tumors with their respective metastasis we found reduced expression in 65% of metastatic tumors compared to primary tumors. In a prior study, we also demonstrated loss of E-cadherin expression in bone metastasis in 24 out of 28 cases (86%).[32] Our results led us to conclude that loss of E-cadherin is a characteristic of progression in prostate cancer. This loss acts altering epithelial cell adhesion, facilitating the invasion of the stroma and the dissemination of the neoplasm.

The loss of E-cadherin may be seen as an alteration related to the epithelial-mesenchymal transition phenomenon.[33] This phenomenon is a biological program required for the acquisition of malignant traits by carcinoma cells and is related to the loss of the cell adhesion associated with the epithelial phenotype. This change leads to the conversion of the tumor cells to a more migratory, mesenchymal-like state and is considered as a necessary step for neoplastic dissemination.

The interaction of catenins with cadherins is a key step in the normal function of adhesion complexes. Alterations in catenin expression can lead to disturbances in cell-cell adhesion, which, in turn, can make the cell more invasive.[34,35] In contrast, in the current study, β-catenin expression was normal in all primary tumors and in 94% of lymph node metastases. Comparing the 17 paired cases, we noticed maintenance of normal expression in all but one case.

In cells lacking E-cadherin, β-catenin is maintained free in the cytoplasm where it can be degraded or transported to the nucleus where it acts as signal transducer promoting transcription of genes associated with cell proliferation; the nuclear expression of β-catenin has been related to worse prognosis.[36,37] In our study, we found normal membranous expression of β-catenin in most cases despite lower E-cadherin expression; this discrepancy may be explained by stabilization of catenins by other molecules as postulated by the Morita study, that found similar discrepancy in 31% of primary prostate cancer evaluated.[14]

γ-catenin down-regulation was observed in almost all cases in our series of prostate cancer metastasis to lymph nodes; only one primary case and two metastases had normal expression, implying that the loss of γ-catenin expression was related to prostate cancer progression.

Although a number of CAM alterations during prostate cancer progression have been described, regulation of these changes is not well understood, and conflicting results have been obtained. Because there have been only a few tissue-based metastatic studies,[38] one can only speculate about the role of integrins and other CAMs in mediating prostate cancer progression, but based on our preliminary results, the loss of CAM seems to be characteristic of this event.

## Conclusions

We found a loss of expression of the majority of integrins, E-cadherin and γ-catenin in primary prostate cancer and in their respective lymph node metastasis, leading us to conclude that the reduction of the expression of these CAMs are important in tumor progression. Further analysis of CAM expression in larger series and studying different stages of tumor development may contribute to the understanding of the role of CAM in the carcinogenesis process.
